# Perspectives on 3D Bioprinting of Peripheral Nerve Conduits

**DOI:** 10.3390/ijms21165792

**Published:** 2020-08-12

**Authors:** Soja Saghar Soman, Sanjairaj Vijayavenkataraman

**Affiliations:** 1 Division of Engineering, New York University Abu Dhabi, Abu Dhabi, UAE; soja.saghar@nyu.edu; 2 Department of Mechanical and Aerospace Engineering, Tandon School of Engineering, New York University, Brooklyn, NY 11201, USA

**Keywords:** peripheral nerve injury, induced pluripotent stem cells (iPSCs), bioprinting, nerve conduits, scaffolds

## Abstract

The peripheral nervous system controls the functions of sensation, movement and motor coordination of the body. Peripheral nerves can get damaged easily by trauma or neurodegenerative diseases. The injury can cause a devastating effect on the affected individual and his aides. Treatment modalities include anti-inflammatory medications, physiotherapy, surgery, nerve grafting and rehabilitation. 3D bioprinted peripheral nerve conduits serve as nerve grafts to fill the gaps of severed nerve bodies. The application of induced pluripotent stem cells, its derivatives and bioprinting are important techniques that come in handy while making living peripheral nerve conduits. The design of nerve conduits and bioprinting require comprehensive information on neural architecture, type of injury, neural supporting cells, scaffold materials to use, neural growth factors to add and to streamline the mechanical properties of the conduit. This paper gives a perspective on the factors to consider while bioprinting the peripheral nerve conduits.

## 1. Introduction

**Figure 1:**
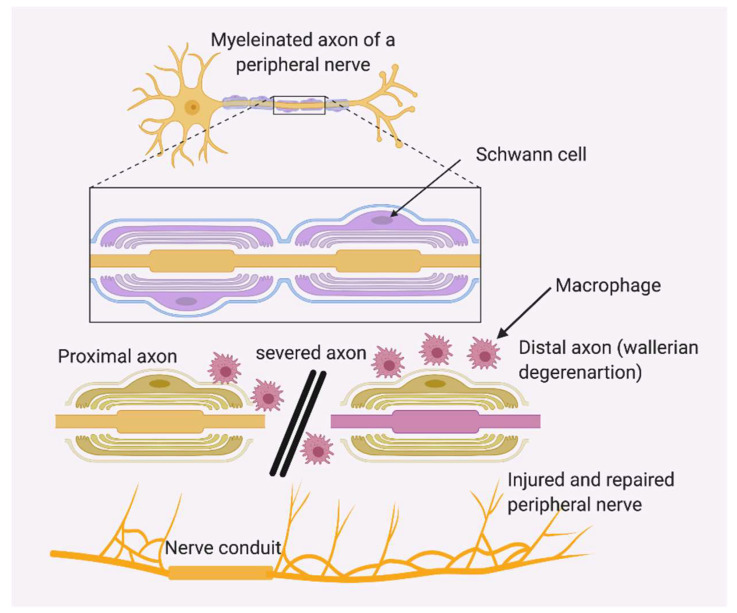
Structure of a peripheral nerve axon. In severe injuries, the axons break apart, and macrophages will clear off the injury site through phagocytosis. A peripheral nerve conduit consisting of supporting cells (Schwann cells, neural progenitor cells, neurons or induced pluripotent stem cells (iPSCs)), growth factors and biocompatible scaffold materials will aid in faster healing and rehabilitation. A nerve conduit used to fill the gap of the injured nerve is an artificially made nerve tissue mimic or artificial nerve graft, which guide axonal regrowth to facilitate regeneration of damaged nerves.

The peripheral nervous system (PNS) consists of a network of 43 pairs of motor and sensory nerves that connect the brain and spinal cord (the central nervous system) to the entire human body. The PNS controls the functions of sensation, movement and motor coordination. The PNS is fragile and can be damaged easily. The incidence of Peripheral Nerve Injury (PNI) is estimated at 18 per 100,000 persons every year in developed countries, whereas it is relatively higher in developing countries [1,2]. In the United States alone, approximately, 20 million people suffer from PNI caused by trauma and neurodegenerative diseases [3]. The nervous system injuries result in an expenditure of around 150 billion US dollars in annual health-care cost in the United States [4,5]. PNI repair is characterized by Wallerian degeneration, axonal growth and remyelination. Seddon in 1972 classified nerve injuries to three categories: neurapraxia without anatomical disruption, which recover within days to weeks; axonotmesis with axonal damage within the nerve, usually recovery takes place within weeks to months; and, the highest degree of damage is neurotmesis, where a nerve is severed, and complete recovery is usually not likely without surgical interventions [6,7]. The complete split in a nerve axon leads to an active degenerative process distal to the injury, known as Wallerian degeneration. The axonal skeleton disintegrates, and the axonal membrane breaks apart in this process, which is followed by degradation of the myelin sheath and infiltration by macrophages. The macrophages clear the debris from the degeneration site. The next step in peripheral nerve injury repair is axonal regeneration. This requires complementary efforts of extracellular matrix molecules released from non-neuronal cells, cytokines and growth factors [8], which is primarily an inflammatory response. The inflammation in the injury site causes axonal disintegration, blood–nerve barrier permeabilization and activation of the peripheral Schwann cells and resident macrophages. Macrophages are the major type of immune cells that carry out phagocytosis until remyelination occurs. The Schwann cells respond to injury by detaching the myelin sheath, cellular proliferation, phagocytosing debris and releasing more cytokines that recruit more inflammatory cells to the tissue damage site (Figure 1).

Most common conditions that require replacement of a piece of peripheral nervous tissue are crushing injuries, penetrating injury, traction injury, ischemia, laceration, compression and burns [9,10,11]. Trauma due to motor accidents, penetrating trauma related to violence, falls and occupational accidents are the most common causes of traumatic injuries to the peripheral nerves [9]. Nerve injuries have devastating consequences on a patient’s quality of life due to sensory and motor function defects, which could be severe enough to paralyze the affected limb, combined with the development of neuropathic pain [12].

Currently, end-to-end neurorrhaphy is considered the clinical gold standard for the treatment of nerve gaps smaller than 1 cm, and autologous nerve grafting is the common treatment for nerve damage exceeding 1 cm [13]. However, limited availability of patient-derived nerve grafts, mismatching size of the nerve tissue, donor site morbidity, possible neuroma formation and adverse immune responses are some of the critical issues limiting autologous nerve grafting as a therapeutic approach [14]. Several alternative approaches have been proposed instead of using patient-derived nerve grafts. Artificially made nerve conduits are an ideal choice for nerve gap filling. 3D printed nerve scaffolds and 3D bioprinted nerve parts offer quick manufacturing of complex peripheral nerve conduits from natural, biological and synthetic biocompatible materials. Bioprinted nerve conduits can serve as an excellent solution for treating such conditions. Additive manufacturing in biology has gained its momentum in recent times, where 3D printing and bioprinting are the integral parts of the process. The swift bioprinting methods allow one to replace the injured nerve tissues with autologous lab-cultured and bioprinted nerve tissues. 3D bioprinting can make peripheral nerve matrices in measured units, using mixtures of biologic, natural and synthetic materials in a bioink. The biological materials in the bioink can be cellular components such as induced pluripotent stem cells, neural stem cells, Schwann cells, astrocytes, glial cells, motor neurons and sensory neurons. The design and bioprinting require comprehensive information on neural architecture, materials to use, supporting cells and growth factors to add as well as streamlining of the mechanical properties of the composite [15]. This brief review explores the components of peripheral nerve conduits and the evolution of 3D bioprinting technologies for the development of peripheral nerve conduits for the management of peripheral nerve injuries.

## 2. Peripheral Nerve Structure

**Figure 2:**
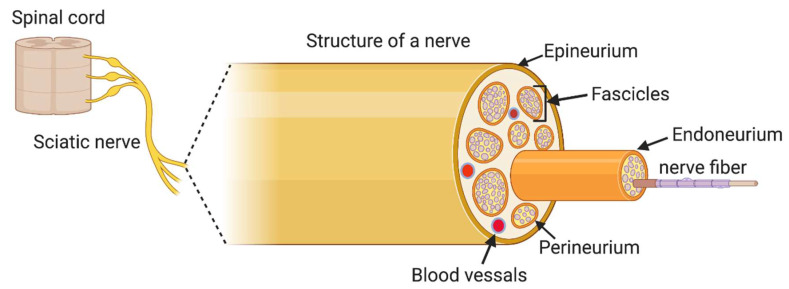
The structure of a peripheral nerve.

The peripheral nervous system is classified into the somatic and autonomic nervous system. Each system consists of afferent (sensory) and efferent (motor) components. A nerve is the primary structure of the PNS that encloses the axons of peripheral neurons, providing a structured pathway that supports neuron function. A nerve consists of structural components including axons, fasciculi, blood vessels like arteries and veins, endoneurial fluid, endoneurium, perineurium and epineurium. The group of neurons inside a nerve is arranged into distinct bundles called fasciculi. Inside each fascicle, neuron cells and blood vessels are held in place by loose connective tissue, termed endoneurium. Each fascicle in a nerve is surrounded by perineurium, which holds the fasciculi together. The number of perineurial cell layers is associated with the diameter of the fascicles; a greater number of fascicles leads to a larger diameter, and a lesser number of fascicles results in a smaller diameter. The layer of dense connective tissue that covers and holds the outer nerve surface is called the epineurium. Blood vessels are located in between each fascicle to exchange nutrients and gases to the neurons. The outer surface of a nerve is covered with epineurium [16]. Exterior to epineurium is the myelin sheath. Schwann cells are a type of glial cell of the peripheral nervous system, located around the neuronal axons, which produce the myelin sheath. When a nerve injury occurs, each of these layers is affected. The axon growth and nerve circuit reorganization are determined by interactions among neurons, Schwann cells and immune cells. These interactions are shaped by growth factors too, which are present in different neuronal compartments. When we design a nerve conduit to treat PNI, we should carefully consider the anatomical features of the nerve tissue (Figure 2) [17]. The ideal scaffold material should support the neural cellular components, augment vascular growth and provide sufficient mechanical strength.

## 3. Supporting Cells

**Figure 3:**
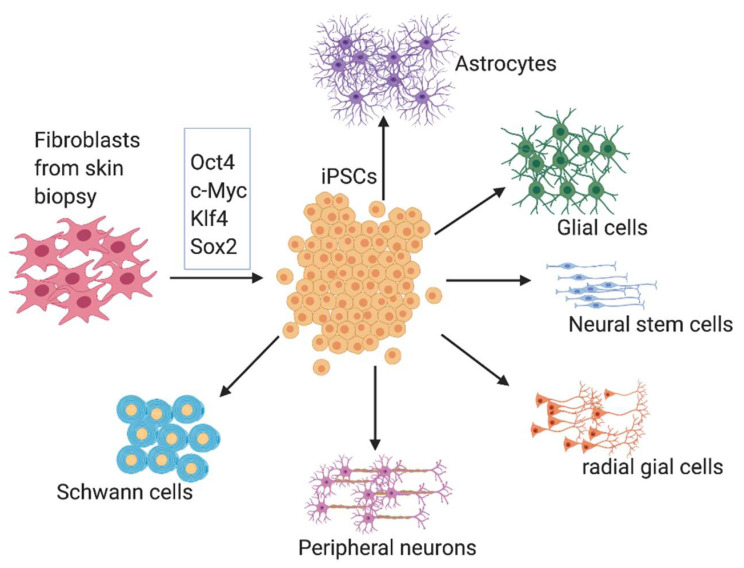
iPSCs serve as a potential source of cellular component for bioprinting. Adult somatic cells collected from peripheral tissues can be de-differentiated to iPSCs. iPSCs can differentiate into different types of cells such as peripheral neurons, radial glial cells, neural stem cells, glial cells and astrocytes using specialized media and growth factors. iPSCs alone or together with other cells, such as Schwann cells or differentiated nerve cells, can be bioprinted along with the scaffold materials. After printing, the bioprinted nerve tissues can be matured in a bioreactor before being transplanted into a peripheral nerve injury site such as a sciatic nerve gap.

Bioprinting requires to use various types of cell sources for developing peripheral nerve conduits. Most bioprinting experiments use different types of stem cells. The stem cell types and their mode of action in the healing process are important to choose the right type of cells for printing. Stem cells are capable to differentiate into Schwann cell-like cells, which can recruit macrophages for damage clearance in the injury site. Stem cells secrete neurotrophic factors to promote the regeneration process [18]. The major sources of supporting cells are induced pluripotent stem cells (iPSCs), embryonic stem cells, neural crest stem cells, mesenchymal stem cells, bone marrow stem cells, adipose-derived stem cells, fetal stem cells, amniotic stem cells, umbilical cord-derived stem cells, Wharton’s jelly-derived stem cells, skin-derived somatic multipotent stem cells, hair follicle-derived stem cells, dental pulp derived stem cells, gingival-derived stem cells and muscle-derived stem cells [19]. Here, we focus on the use of iPSCs and their derivatives as the cell source for bioprinting since iPSCs are the most promising cells given their versatility and availability as an autologous source (Figure 3).

### 3.1. Induced Pluripotent Stem Cells (iPSCs)

Shinya Yamanaka and Takahashi in 2006 triggered a revolution in the field of stem cell biology by discovering the technique to produce induced pluripotent stem cells (iPSCs) [20]. As a consequence, an entire biomedical industry has developed around the promise of using human iPSCs and their derivatives for cell therapy-based transplantation medicine. The protocols for differentiation of neural lineages are well established by many laboratories all over the world [21,22]. iPSCs have proven to be useful in aiding the regeneration of peripheral nerve injury in animal models [23]. Peripheral nerve gaps in mice were repaired using tissue-engineered bioabsorbable nerve conduits consisting of l-lactide and ε-caprolactone coated with iPSC-derived neurospheres. In this study, the nerve conduit lumen wall was engineered into two-layers. The inner layer was made of 50% poly l-lactide sponge copolymer and 50% poly ε-caprolactone (PCL). These scaffold materials were flexible, biocompatible and bioabsorbable. The inner layer was designed to have a honeycomb-like structure containing pores of 10–50 μm into which cells can enter and proliferate. The outer layer of the conduit was made of poly l-lactide multifilament fiber mesh to strengthen the tube. The neurospheres derived from mouse iPSCs were used as the cellular component to make the nerve conduit. A dense number of cells, around 4 × 10^6^ cells were suspended to make each conduit and 3D-cultured for 14 days before being implanted in 5 mm sciatic nerve gaps in mice. Motor and sensory recovery was better in the iPSC conduit treated group of mice after 4, 8 and 12 weeks. At 12 weeks, all the nerve conduits remained structurally stable without any collapse, and histological analysis indicated axonal regeneration in the nerve conduits of both groups [24]. The iPSCs in the 3D culture formed neurospheres differentiated to neural or glial cells [25,26]. In another study, sciatic nerve gaps in mice were reconstructed using a nerve conduit made up of a combination of iPSC-derived neurospheres and a basic fibroblast growth factor (bFGF)-incorporated gelatin microsphere drug delivery system. The tissue-engineered nerve conduit consists of iPSCs and bioabsorbable scaffolds, and a bFGF drug delivery system was found to be very effective in peripheral nerve regenerative therapy [27,28].

Though iPSCs are the popular choice over other types of stem cells for making nerve conduits, considering the ethical compliance and immuno-compatibility, there are still concerns over the use of iPSCs in extensive clinical applications. iPSCs can induce tumors due to chromosomal aberrations and epigenetic alterations [29]. There is more research work to be done in these areas to develop foot-print free and xeno-free iPSCs and their derivatives for use in clinics. Incomplete de-differentiation of iPSCs is another challenge to be addressed. The cells used for clinical applications should be pure populations of specific cells.

### 3.2. iPSC-Derived Neural Crest Stem Cells

Neural crest stem cells represent an intermediary multipotent cell population that could be differentiated further to numerous anatomical structures such as the peripheral nervous system, dental structures and cornea [30]. Purified iPSC-derived neural crest stem cells were used for treating a sciatic nerve defect model in mice. Neural crest cells with low-affinity nerve growth factor receptor (LNGFR)—and thymocyte antigen-1 (THY-1)—positive (LT-NCLCs) were derived from human iPSCs. The murine sciatic nerve defect model was treated with a bridging silicone tube prefilled with LT-NCLCs and showed increased myelination, angiogenesis and motor function recovery as compared to the control group. LT-NCLCs promoted axonal regrowth and remyelination by recruiting Schwann cells. Transplantation of LT-NCLCs is a promising approach for nerve regeneration treatment of massive peripheral nerve defects [31]. Neural crest cells (NCCs) derived from the ectoderm could differentiate into neural lineage cells, including Schwann cells. Previous studies have reported the effectiveness of transplanting iPSC-derived NCCs for nerve regeneration [32,33,34]. The impact of neural crest stem cell transplantation in the treatment of peripheral nerve injury mainly depends on their capacity to differentiate to other neural cells, secretion of neurotrophins and promotion of new myelin formation.

The complete mechanistic cues leading to peripheral nerve injury repair are still unclear. Neural crest stem cells (NCSCs) can be used as an effective stem cell component while devising the nerve conduit for peripheral nerve repair. It has been reported that implantation of neural crest-derived multipotent stem cells in a sciatic nerve transection model in adult Friend Virus B (FVB) strain mice promoted a higher rate of nerve regeneration compared with that in mice without transplantation. The work showed the role of NCSCs in promoting nerve injury repair by differentiating into Schwann cells. The intensity of vascularization and renewal of the endoneurium also increased after NCSC transplantation [35]. Peripheral nerve injuries could be accompanied by skin injuries. 3D skin reconstructions innervated using NCSCs would be a feasible approach to develop and restore the sensory and motor functions of skin [36].

### 3.3. Schwann Cells

Transplantation of lab-differentiated Schwann cells not only provides support for axon migration but also secretes neurotrophic factors to promote nerve growth [37,38,39]. Schwann cells differentiate and enhance the nerve regrowth pathways, and they secrete myelin around axons in the injured peripheral nerves. Bioprinting of nerve scaffolds incorporating Schwann cells is a chosen method over the use of autografts, due to the difficulty in obtaining the autografts and the effectiveness of the cell-incorporated live nerve conduits. Schwann cells were suspended in hydrogel mixtures comprising alginate, fibrinogen, thrombin, hyaluronic acid (HA) and RGD (Arg-Gly-Asp) peptides as scaffold materials and bioprinted with the appropriate crosslinking agents to produce nerve scaffolds. After bioprinting, the performance of Schwann cells within scaffolds and the orientation and elongation of neurons seeded on the printed scaffolds were examined and analyzed for the potential applications in nerve tissue regeneration [40]. Living Schwann cells encapsulated within the scaffolds after biofabrication were viable and proliferate in culture. Transplantation of the cultured bioprinted conduit provided haptotactic cues directing alignment of encapsulated Schwann cells and elongation of dorsal root ganglion neurites along the 3D-printed strands for guided nerve growth [41]. In 2017, Kern et al. reported a method to obtain human iPSC-derived Schwann cells. The cells were generated through dual SMAD inhibition and isolated by FACS [42]. The precursor CD49d+ cells were isolated and used for Schwann cell differentiation. The Schwann cells combined with growth factor GDNF and chondroitinase in fibrin gel showed high axonal count and myelination in a rat hindlimb nerve injury model. iPSC-derived Schwann cells are available commercially, which can be used for bioprinting. One such cell is Tempo-iSchwann™ cells, which are reprogrammed from footprint-free human iPSCs [43]. They can be co-cultured with neurons, and they express mRNA biomarkers for Schwann cells: *GAP43, MPZ, NCAM1, NGFR, OCT6, Sox2* and *Sox10*. They also express S100 β and MBP in immunocytochemistry. In 2019, Carrio et al. generated iPSCs from neurofibromatosis type 1 plexiform neurofibroma (PNF) cells, and those iPSCs were differentiated into neural crest cells and Schwann cells. For generating neural crest cells, they employ chemically defined medium to activate Wnt signaling while inhibiting Activin/Nodal/transforming growth factor β signaling [44,45,46], and Schwann cells were differentiated from the neural crest cells.

## 4. Scaffold Materials

Restoring peripheral nerve damage is a challenging task. Polymers are the favorite choice as a nerve scaffold material. Histocompatibility and mechano-compatibility of the scaffold are important while printing a nerve guidance conduit. Both synthetic and natural polymers are extensively tested as scaffold materials for restoring functions in injured peripheral nerves. Polymers differ in their physical, chemical, mechanical and inherent biological properties. Polymers are selected based on the end uses such as hydrogel applications, drug delivery vehicles, 3D cell culture, nerve conduits and tissue scaffolds. Successful polymers chosen for nerve conduit printing should offer mechanical support for growing neurites, reduce scar tissue formation, regulate cell healing signals to guide axonal growth and augment tissue regeneration and integration [47,48]. Polymeric neural probes and electrodes made with graphene nanoparticles are successfully implanted to treat peripheral nerve injury conditions and neurodegenerative diseases in animal models [49,50,51,52]. Several studies have shown the use of polymers as nerve support structures and nerve conduits, which improved the regeneration of damaged neural tissues.

In general, natural polymers or bio-derived polymers, isolated from nature, offer the advantage of better biocompatibility and bioactivity, while synthetic polymers show better resilience and structural stability. The combined use of both natural and synthetic polymers in a nerve conduit would mimic the native physiological environment of healthy neural tissues and effectively regulate cell growth, remyelination and repairing of injured nerve tissues. Some of the natural polymers are water-soluble, and they can dissolve in cell culture media and saline. Examples of such polymers are gelatin, alginate, fibrinogen and hyaluronic acid. They can form solutions and hydrogels, which help to retain the fluidity to 3D print in layers [53,54,55]. During initial printing using a bioprinter, the flow behavior of the prepared hydrogel solution can be evaluated and based on the performance of the hydrogel. Bioprinting parameters, including the dispensing pressure, speed of the dispensing head and concentration of crosslinkers, could be identified and used to create scaffolds with designed structures. By adjusting the fluid-gel state, the live cell components can be suspended in the hydrogels for printing and post-printing processes. In addition, it is easy to incorporate growth factors in the hydrogel scaffolds. The hydrogel scaffold should mimic the biologic environment and facilitate cellular attachment, proliferation and growth, aid the dispersion of bioactive molecules and growth factors, and it should contain space for accommodating the extracellular matrix. Natural polymers with hydrogel consistency can be printed using a bioprinter in a layer-by-layer fashion. Once the printing is over, most of the biopolymers need cross-linking using physical or/and chemical methods to provide stability for the printed structure. Glutaraldehyde, calcium chloride, thrombin and UV light are the crosslinker choices for natural polymers. When we choose the method of crosslinking, it should be mild and compatible with live cells [56,57,58]. The peripheral nerve conduits require sturdier architecture than the soft parenchymatous organs. The post-printing processes of the tissues should avoid extreme temperature variations, organic solvents and dehydration, which can kill the cellular components and reduce the activity of the biomolecules incorporated [59,60,61]. While 3D bioprinting, some of the scaffold material, such as fibrin-based hydrogels incorporated with cellular components, could be challenging to work with due to the mechanical incompatibilities caused by low viscosity [62]. Other natural scaffold materials are chitosan, silk fibroin and hyaluronic acid (HA). HA is a natural extracellular matrix component, used extensively in 3D bioprinting. HA is a long-chain sugar-like molecule well known for inducing wound healing and nerve regeneration. HA is an excellent biomaterial candidate for nerve conduits [63,64].

Assessing peripheral nerve injuries and devising suitable treatment modalities are occlusive most of the time in the personalized medicine field. Physicians and scientists have to carefully consider the extent of the injury on a large scale as well as the cellular aspects before using a nerve conduit. It should be biocompatible, biodegradable and possess enough strength to stay intact for a certain period of time until the nerve gaps are filled with newly formed axons. The synthetic polymers and its mixtures with natural polymers serve suitable roles in bioprinting such nerve conduits. In particular, polyurethane, Polypyrrole (PPy)/Chitosan, poly (phosphoester) [65,66], collagen [67,68], polyglycolide [69], collagen and poly-glycolide [70], poly (l-lactide-co-glycolide) (PLGA), poly-l-lactic acid/caprolactone and PCL/PPy Conductive Scaffolds [71] are among the most common polymers or mixtures used for this purpose. Nerves are electrophysiological organs; hence, nerve conduits with electrical conductivity would go a long way to help regain the complete functionality of nerves. Graphene and carbon nanotubes have been used as biocompatible, non-immunogenic conductive composite materials in experimental nerve conduit models [72]. Gelatin- and graphene-incorporated nerve guidance conduits were printed to make conductive conduits [73]. The 3D-printed conductive block copolymer of PPy and PCL (PPy-b-PCL), and pure PCL nerve conduits were evaluated in vitro. The growth and differentiation of human embryonic stem cell-derived neural crest stem cells (hESC-NCSCs) to peripheral neurons were investigated in these scaffolds. PCL/PPy scaffolds supported higher growth of neural cells and a stronger maturation of hESC-NCSCs to peripheral neuronal cells [74]. The addition of nerve growth factor (NGF) in hydrogels has shown to improve neuron growth and promote their development, differentiation, growth, regeneration and other functional properties. NGF induced directional growth and enhanced the differentiation of Schwann cells in co-culture systems [75]. Along with the structure and biological compatibility, the 3D-printed nerve conduit should be able to withstand the tensions and shear forces induced during and after the surgical procedures. The conduit should closely replicate the mechanical properties of the native nerve tissue. To assess the bioprinted conduit for mechanical strength, physical parameters such as the Young’s modulus, compressive strength, suture retention strength and ultimate tensile strength have to be measured [76,77,78]. Rat sciatic nerve defect models are the gold standard for testing nerve conduits, but the parameters of rat models would not directly translate to human conditions. Human peripheral nerves, such as the ulnar and median nerves, show an elastic modulus falling within 10–20 kPa [79], while that of rat sciatic nerve shows Young’s modulus of 580 kPa and ultimate stress of about 2720 kPa [80]. By choosing the right printing materials and printing parameters, biomimicry could be achieved in the fabricated conduits.

## 5. Bioprinting of Nerve Conduits

**Figure 4:**
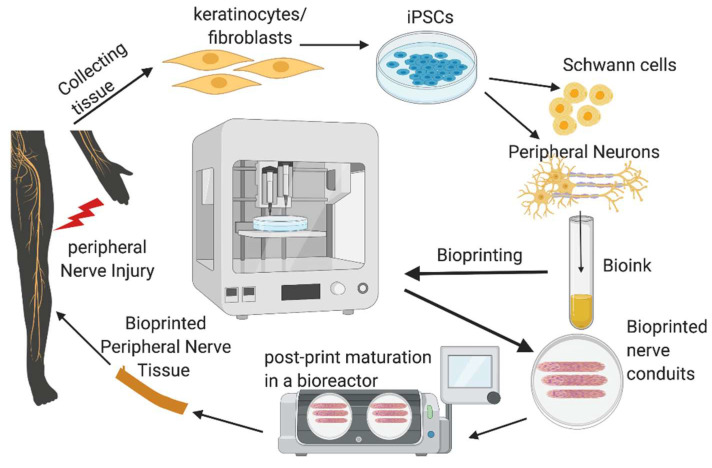
Bioprinting of nerve conduits. The peripheral cells, like keratinocytes or fibroblasts, are collected from the skin, and pluripotency is induced in these cells to make iPSCs. The iPSCs are differentiated to peripheral neurons or iPSC-cells as such will be suspended in the scaffold material to make the bioink. The nerve conduit would then be printed using a suitable bioprinter, post-processed and used for transplantation in the peripheral nerve injury site.

Development of peripheral nerve conduits is an emerging area of considerable attention in both neurobiology and bioprinting research, aiming to promote healing of severed peripheral nerves. An ideal nerve guide conduit (NGC) should mimic the biological and physical attributes of the targeted nerve. It should include the topographic axon guidance cues, cellular and non-cellular components embedded as part of their composition. The past 10 years witnessed tremendous growth in the development of NGCs using novel scaffold materials, iPSCs and iPSC-derived cell components, adding new growth factors, drug molecules and nanomaterials that promote guided nerve regeneration to aid in nerve repair (Table 1). Bioprinting uses cells as building blocks and scaffold materials as a matrix to generate 3D tissue constructs. The preparation of bioink for bioprinting requires the addition of living cells in a controlled manner in order to construct nerve parts that closely mimic natural tissues. The bioink is deposited on a surface in a 3D manner using a computer software-controlled bioprinter within a specified time in a biosafety cabinet with controlled temperature and humidity. The deposited tissue material is subjected to post-print processing such as crosslinking to stabilize the printed structure [81,82]. Electrohydrodynamic jet (EHD-jet) 3D printing, extrusion-based printing, melt-electrospinning writing, inkjet bioprinting, laser-assisted bioprinting, the Kenzan method, and stereolithography-based methods are some of the bioprinting methods used for printing nerve conduits [83,84,85,86] (Figure 4). Among these different printing techniques, extrusion and electro-hydrodynamic jet printing appear to be promising for stem cell printing. The methods without harsh treatments are preferable while using highly sensitive cell populations such as iPSCs and embryonic stem cells. Therefore, printing methods using UV radiation, photo-crosslinking, lasers and high pressure are least preferred while printing nerve conduits. Many studies have used scaffold-free bioprinting of nerve cells for making nerve conduits. However, in order to assure the stability of the conduit in the injured site, and to augment complete healing of injury, inclusion of various fibrous scaffold materials in the NGCs is required. Nerve conduits with a fine structure on micro and nano scales have been printed and used to create artificial nerve fibers, and they have been successful in healing around 30 mm nerve gaps in a rat model of sciatic nerve injury [87].

Human iPSC-derived cortical neurons and precursor glial cells were 3D bioprinted using the extrusion method. The printing experiment proved to have no adverse effect on cell viability in the short term and long term, until 70 days. The bioprinted neural construct expressed neuronal markers and neural network activities in specified time points of differentiation. Alginate and Matrigel were used as the bioink and calcium chloride as the cross-linker. The induction of cortical neurons from iPSC-derived cells was done by dual SMAD and Hedgehog signaling inhibition with cyclopamine [88]. In 2013, Owens et al. bioprinted a cellular nerve conduit using bone marrow stem cells and Schwann cells and tested the bioprinted graft in a sciatic nerve injury mice model. They used agarose rods to stabilize the cellular structure in a layer-by-layer fashion. The conduit showed signs of promising functional activity and axonal growth augmenting nerve injury repair [90]. 3D porous nerve guide conduits were printed using an electrohydrodynamic jet 3D printing process using a biodegradable and conductive block copolymer of PPy and PCL (PPy-b-PCL) scaffold material. Human embryonic derived neural crest stem cells were grown on these conduits and differentiated to peripheral neurons. The PCL/PPy scaffolds enhanced the growth and differentiation of neural cells to peripheral neuronal cells. The speed and accuracy of bioprinting have improved in recent years. Vega et al. demonstrated bioprinting of human iPSC-derived neural progenitor cells into a neural tissue structure with a unique fibrin-based bioink, which takes less than 5 min to print four tissue structures [91,93]. The cell-friendly printing process was attributed to a high cell component viability up to >81% live cells in the printed tissue. Recently, 4D bioprinting is being explored to achieve desirable shapes and mechanical properties post-printing. The printed structure can fold, curl, twist and expand using different materials joined by hinges based on specific physiological stimuli. Maio et al. developed a bioprinted proof-of-concept 4D nerve conduit for repairing peripheral nerve injury [92]. Considering the complexity of the nervous system tissues, only a few studies have addressed the bioprinting of NGCs. Streamlining the bioprinting of NGCs using iPSCs, neural stem cells and differentiated nerve cells is a developing field in regenerative medicine. More research needs to be done on the types of cells to be used in the bioinks, growth factors suitable for cell differentiation, types of hydrogels to be used, printing conditions and on the overall methodology of bioprinting [94].

## 6. Summary and Future Perspectives

Peripheral nerve tissues possess an intricate architecture with complex anatomical features. In conditions where nerves get damaged by severance injury, nerve conduits are essential to bridge the gap. The choice of regenerative scaffold materials has to provide the correct microstructure in order to guide the neural cell orientation in the repairing process. Bioprinting allows the production of nerve conduits with highly precise spatial arrangement of cells, biomaterials and nerve growth factors. An ideal 3D bioprinted conduit should mimic the exact structural details of the region of the nerve to be replaced, to facilitate the nerve growth guidance for regeneration and integration of the conduit into the severed nerve body. Bioprinting of nerve conduit offers great promise, yet there are many technical difficulties to achieve the correct architecture of the real nerve tissue in terms of nerve micro-anatomy, deposition of different types of cells in a spatially controlled manner in the hydrogel, composition and mechanical properties of the bioink, vascularization and innervation of the transplanted nerve tissues. Another area to be explored is the in situ printing of the nerve conduit to the injured sites. Integrating new developing fields such as Artificial Intelligence and Machine Learning (AI-ML) along with bioprinting can help in devising in situ bioprinting techniques, which in due course can avoid prolonged periods of scaffold preparation and cell handling outside the body. Moreover, AI-ML-enabled bioprinting can aid to improve efficiency in pre-printing processing, defect detection and real-time control while printing and predicting the maintenance in different stages of bioprinting.

Another interesting area of research in bioprinting is the design novel bioinks. Improved bioinks comprising supporting cells, growth-promoting biomolecules and biocompatible, biodegradable, cell-friendly hydrogels possessing appropriate mechanical strength are in high demand. Bioprinted nerve tissues are not only expected to support the nerve growth but also sustain the mechanical stresses associated with the injury site during surgery as well as during normal physical movements. Hydrogels, being soft and having weak mechanical properties, have to be tuned in composition to enhance the strength and post-printing structural stability by incorporation of nanoparticles, composite bioink approaches and adopting suitable cross-linking mechanisms. In addition, the bioinks should not cause any unfavorable immune reaction but encourage the healing process once transplanted. Investigations on biocompatible, least-immunogenic composite bioinks with appropriate mechanical properties, incorporation of functional materials such as conductive inks, 4D bioprinting and in situ bioprinting are some of the future areas of research in bioprinted nerve conduits. Collecting, culturing and bioprinting patient-derived iPSCs and differentiated nerve precursor cells are other areas that the bioprinting field should focus on. With the right functional bioinks, appropriate bioprinting techniques, iPSC technology and an optimized fabrication of biomimetic nerve conduits comprising sourcing patient cells, inducing pluripotency, formulating the bioink, bioprinting the conduit and post-printing maturation, clinical translation of bioprinted nerve conduits could be realized.

## Figures and Tables

**Table 1:** Bioprinting of nerve conduits.

Cells Used	Type of Bioprinting	Type of Cells/Tissue Produced	Bioink or Scaffold Used	Cross-linker Used, and Conditions	References	Representative Images
Human iPSC derived cortical neurons and precursor glial cells	Extrusion	3D neuronal construct	2% *w/v* alginate and 0.5 × Matrigel (~50% dilution from stock)	Calcium Chloride (80 mM)	[88,89]	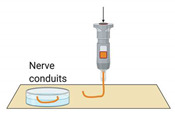
Mouse bone marrow stem cells and Schwann cells	Extrusion	Sciatic nerve conduit	Agarose rods, self-assembled cellular bioink	Temperature (below 40 °C)	[90]	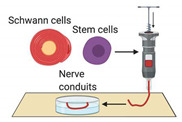
Human embryonic derived neural crest stem cells	Electro-hydrodynamic jet printing	Peripheral neuronal cells	PPy and Polycaprolactone (PPy-b-PCL) scaffold	Temperature (below 50 °C)	[74]	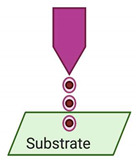
Human iPSC derived neural progenitor cells	Microfluidic Lab-on-a-Printer (LOP)	Neural progenitor cell cylindrical construct	Fibrinogen, Alginate	Calcium chloride (20 mg/mL), chitosan (0.075% *w*/*v*), thrombin (1.7 U/mL)	[91]	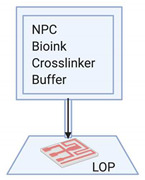
NT2 cells	Inkjet printing	Neural sheets	Fibrin gels	Calcium chloride (20 μm), Bovine thrombin (20 IU/mL)	[83]	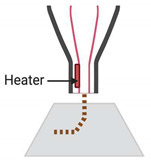
Human mesenchymal stem cells	Stereolithography	Nerve guidance conduit	Graphene Nanohybrid, soybean oil epoxidized acrylate	UV light (355 nm)	[92]	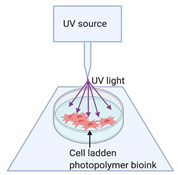

## References

[1] Thomson J.A. (1998). Embryonic Stem Cell Lines Derived from Human Blastocysts. Science.

[2] Martens W., Bronckaers A., Politis C., Jacobs R., Lambrichts I. (2013). Dental stem cells and their promising role in neural regeneration: An update. Clin. Oral Investig..

[3] Lundborg G., Richard P. (2003). Bunge memorial lecture. Nerve injury and repair—A challenge to the plastic brain. J. Peripher. Nerv. Syst..

[4] Taylor C.A., Braza D., Rice J.B., Dillingham T. (2008). The incidence of peripheral nerve injury in extremity trauma. Am. J. Phys. Med. Rehabil..

[5] Grinsell D., Keating C.P. (2014). Peripheral nerve reconstruction after injury: A review of clinical and experimental therapies. Biomed. Res. Int..

[6] Kaya Y., Sarikcioglu L. (2015). Sir Herbert Seddon (1903–1977) and his classification scheme for peripheral nerve injury. Childs Nerv. Syst..

[7] Lee S.K., Wolfe S.W. (2000). Peripheral nerve injury and repair. J. Am. Acad. Orthop. Surg..

[8] Gaudet A.D., Popovich P.G., Ramer M.S. (2011). Wallerian degeneration: Gaining perspective on inflammatory events after peripheral nerve injury. J. Neuroinflamm..

[9] Diogo C.C., Camassa J.A., Pereira J.E., Da Costa L.M., Filipe V., Couto P.A., Geuna S., Maurício A.C., Varejão A.S.P. (2017). The use of sheep as a model for studying peripheral nerve regeneration following nerve injury: Review of the literature. Neurol. Res..

[10] Reichert P., Wnukiewicz W., Witkowski J., Bocheńska A., Mizia S., Gosk J., Zimmer K. (2016). Causes of Secondary Radial Nerve Palsy and Results of Treatment. Med. Sci. Monit..

[11] Adigüzel E., Yaşar E., Tecer D., Güzelküçük Ü., Taskaynatan M.A., Kesikburun S., Ozgul A. (2016). Peripheral nerve injuries: Long term follow-up results of rehabilitation. J. Back Musculoskelet Rehabil..

[12] Kouyoumdjian J., Graç C., Ferreira V.F.M. (2017). Peripheral nerve injuries: A retrospective survey of 1124 cases. Neurol. India.

[13] Siemionow M., Brzezicki G. (2009). Chapter 8: Current techniques and concepts in peripheral nerve repair. Int. Rev. Neurobiol..

[14] Johnson E.O., Zoubos A.B., Soucacos P.N. (2005). Regeneration and repair of peripheral nerves. Injury.

[15] Philips C., Cornelissen M., Carriel V., Cornelissen R. (2018). Evaluation methods as quality control in the generation of decellularized peripheral nerve allografts. J. Neural Eng..

[16] Dzobo K., Thomford N.E., Senthebane D.A., Shipanga H., Rowe A., Dandara C., Pillay M., Motaung K.S.C.M. (2018). Advances in Regenerative Medicine and Tissue Engineering: Innovation and Transformation of Medicine. Stem Cells Int..

[17] Braga-Silva J., Marchese G., Cauduro C.G.D.S., DeBiasi M. (2017). Nerve conduits for treating peripheral nerve injuries: A systematic literature review. Hand Surg. Rehabil..

[18] Evans G.R. (2001). Peripheral nerve injury: A review and approach to tissue engineered constructs. Anat. Rec..

[19] Sullivan R., Dailey T., Duncan K., Abel N., Borlongan C.V. (2016). Peripheral Nerve Injury: Stem Cell Therapy and Peripheral Nerve Transfer. Int. J. Mol. Sci..

[20] Jiang L., Jones S., Jia X. (2017). Stem Cell Transplantation for Peripheral Nerve Regeneration: Current Options and Opportunities. Int. J. Mol. Sci..

[21] Takahashi K., Yamanaka S. (2006). Induction of pluripotent stem cells from mouse embryonic and adult fibroblast cultures by defined factors. Cell.

[22] Denham M., Dottori M. (2011). Neural differentiation of induced pluripotent stem cells. Methods Mol. Biol..

[23] Hu B., Weick J.P., Yu J., Ma L., Zhang X., Thomson J.A., Zhang S.-C. (2010). Neural differentiation of human induced pluripotent stem cells follows developmental principles but with variable potency. Proc. Natl. Acad. Sci. USA.

[24] Ikeda M., Uemura T., Takamatsu K., Okada M., Kazuki K., Tabata Y., Ikada Y., Nakamura H. (2014). Acceleration of peripheral nerve regeneration using nerve conduits in combination with induced pluripotent stem cell technology and a basic fibroblast growth factor drug delivery system. J. Biomed. Mater. Res. A.

[25] Uemura T., Takamatsu K., Ikeda M., Okada M., Kazuki K., Ikada Y., Nakamura H. (2012). Transplantation of induced pluripotent stem cell-derived neurospheres for peripheral nerve repair. Biochem. Biophys. Res. Commun..

[26] Uemura T., Takamatsu K., Ikeda M., Okada M., Kazuki K., Ikada Y., Nakamura H. (2011). A tissue-engineered bioabsorbable nerve conduit created by three-dimensional culture of induced pluripotent stem cell-derived neurospheres. Biomed. Mater. Eng..

[27] Uemura T., Ikeda M., Takamatsu K., Yokoi T., Okada M., Nakamura H. (2014). Long-term efficacy and safety outcomes of transplantation of induced pluripotent stem cell-derived neurospheres with bioabsorbable nerve conduits for peripheral nerve regeneration in mice. Cells Tissues Organs.

[28] Nectow A.R., Marra K.G., Kaplan D.L. (2012). Biomaterials for the development of peripheral nerve guidance conduits. Tissue Eng. Part B Rev..

[29] Ben-David U., Benvenisty N. (2011). The tumorigenicity of human embryonic and induced pluripotent stem cells. Nat. Rev. Cancer.

[30] Munoz W.A., Trainor P. (2015). Neural crest cell evolution: How and when did a neural crest cell become a neural crest cell. Curr. Top Dev. Biol..

[31] Kimura H., Ouchi T., Shibata S., Amemiya T., Nagoshi N., Nakagawa T., Matsumoto M., Okano H., Nakamura M., Sato K. (2018). Stem cells purified from human induced pluripotent stem cell-derived neural crest-like cells promote peripheral nerve regeneration. Sci. Rep..

[32] Lv Y., Nan P., Chen G., Sha Y., Xia B., Yang L. (2015). In vivo repair of rat transected sciatic nerve by low-intensity pulsed ultrasound and induced pluripotent stem cells-derived neural crest stem cells. Biotechnol. Lett..

[33] Okawa T., Kamiya H., Himeno T., Kato J., Seino Y., Fujiya A., Kondo M., Tsunekawa S., Naruse K., Hamada Y. (2013). Transplantation of neural crest-like cells derived from induced pluripotent stem cells improves diabetic polyneuropathy in mice. Cell Transplant..

[34] Huang C.-W., Huang W.-C., Qiu X., Da Silva F.F.F., Wang A., Patel S., Nesti L.J., Poo M.-M., Li S. (2017). The Differentiation Stage of Transplanted Stem Cells Modulates Nerve Regeneration. Sci. Rep..

[35] Vasyliev R.G., Rodnichenko A.E., Shamalo S.N., Demidchouk A.S., Labunets I.F., Chaikovskii Y.B., Butenko G.M. (2015). Effects of Neural Crest-Derived Multipotent Stem Cells on Regeneration of an Injured Peripheral Nerve in Mice. Neurophysiology.

[36] Biedermann T., Böttcher-Haberzeth S., Klar A.S., Pontiggia L., Schiestl C., Meuli-Simmen C., Reichmann E., Meuli M. (2013). Rebuild, restore, reinnervate: Do human tissue engineered dermo-epidermal skin analogs attract host nerve fibers for innervation?. Pediatr. Surg. Int..

[37] Yu Z., Men Y., Dong P. (2017). Schwann cells promote the capability of neural stem cells to differentiate into neurons and secret neurotrophic factors. Exp. Ther. Med..

[38] Jessen K.R., Mirsky R., Lloyd A.C. (2015). Schwann Cells: Development and Role in Nerve Repair. Cold Spring Harb. Perspect Biol..

[39] Namgung U. (2014). The role of Schwann cell-axon interaction in peripheral nerve regeneration. Cells Tissues Organs.

[40] Ning L., Sun H., Lelong T., Guilloteau R., Zhu N., Schreyer D.J., Chen X., Chen D.X. (2018). 3D bioprinting of scaffolds with living Schwann cells for potential nerve tissue engineering applications. Biofabrication.

[41] England S., Rajaram A., Schreyer D.J., Chen X. (2017). Bioprinted fibrin-factor xiii-hyaluronate hydrogel scaffolds with encapsulated schwann cells and their in vitro characterization for use in nerve regeneration. Bioprinting.

[42] Kern B., Sarhane K., Ibrahim Z., Mukherjee-Clavin B., Budihardjo J., Cashman C., Krick K., Schneeberger S., Lee W., Mao H.-Q. (2017). Induced Pluripotent Stem Cell (iPS) Derived Schwann Cells to Enhance Functional Recovery Following Nerve Injury and Limb Allotransplantation. Am. J. Transplant..

[43] https://www.tempobioscience.com/products/cell-models/tempo-ischwann.html.

[44] Carrió M., Mazuelas H., Richaud-Patin Y., Gel B., Terribas E., Rosas I., Jimenez-Delgado S., Biayna J., Vendredy L., Blanco I. (2019). Reprogramming Captures the Genetic and Tumorigenic Properties of Neurofibromatosis Type 1 Plexiform Neurofibromas. Stem Cell Rep..

[45] Lee H.-Y., Kléber M., Hari L., Brault V., Suter U., Taketo M.M., Kemler R., Sommer L. (2004). Instructive role of Wnt/beta-catenin in sensory fate specification in neural crest stem cells. Science.

[46] Gomez G.A., Prasad M.S., Sandhu N., Shelar P.B., Leung A.W., García-Castro M.I. (2019). Human neural crest induction by temporal modulation of WNT activation. Dev. Biol..

[47] Willerth S.M., Sakiyama-Elbert S.E. (2007). Approaches to neural tissue engineering using scaffolds for drug delivery. Adv. Drug Deliv. Rev..

[48] Ashraf R., Sofi H.S., Beigh M.A., Majeed S., Arjamand S., Sheikh F.A. (2018). Prospects of Natural Polymeric Scaffolds in Peripheral Nerve Tissue-Regeneration. Adv. Exp. Med. Biol..

[49] Castagnola V., Descamps E., Lecestre A., Dahan L., Remaud J., Nowak L., Bergaud C. (2015). Parylene-based flexible neural probes with PEDOT coated surface for brain stimulation and recording. Biosens. Bioelectron..

[50] Boni R., Ali M.A., Shavandi A., Clarkson A.N. (2018). Current and novel polymeric biomaterials for neural tissue engineering. J. Biomed. Sci..

[51] Uz M., Donta M., Mededovic M., Sakaguchi D.S., Mallapragada S.K. (2019). Development of Gelatin and Graphene-Based Nerve Regeneration Conduits Using Three-Dimensional (3D) Printing Strategies for Electrical Transdifferentiation of Mesenchymal Stem Cells. Ind. Eng. Chem. Res..

[52] Bonandrini B., Figliuzzi M., Papadimou E., Morigi M., Perico N., Casiraghi F., Sangalli F., Conti S., Benigni A., Remuzzi A. (2014). Recellularization of well-preserved acellular kidney scaffold using embryonic stem cells. Tissue Eng. Part A.

[53] Ozbolat I.T., Hospodiuk M. (2016). Current advances and future perspectives in extrusion-based bioprinting. Biomaterials.

[54] Matai I., Kaur G., Seyedsalehi A., McClinton A., Laurencin C.T. (2020). Progress in 3D bioprinting technology for tissue/organ regenerative engineering. Biomaterials.

[55] Zhang Y.S., Yue K., Aleman J., Mollazadeh-Moghaddam K., Bakht S.M., Yang J., Jia W., Dell’Erba V., Assawes P., Shin S.R. (2017). 3D Bioprinting for Tissue and Organ Fabrication. Ann. Biomed. Eng..

[56] Wang X. (2012). Intelligent freeform manufacturing of complex organs. Artif. Organs.

[57] Derby B. (2012). Printing and prototyping of tissues and scaffolds. Science.

[58] Pişkin E. (1995). Biodegradable polymers as biomaterials. J. Biomater. Sci. Polym. Ed..

[59] Toh W.S., Loh X.J. (2014). Advances in hydrogel delivery systems for tissue regeneration. Mater. Sci. Eng. C Mater. Biol. Appl..

[60] Billiet T., Vandenhaute M., Schelfhout J., Van Vlierberghe S., Dubruel P. (2012). A review of trends and limitations in hydrogel-rapid prototyping for tissue engineering. Biomaterials.

[61] Hou R., Nie L., Du G., Xiong X., Fu J. (2015). Natural polysaccharides promote chondrocyte adhesion and proliferation on magnetic nanoparticle/PVA composite hydrogels. Colloids Surf. B Biointerfaces.

[62] Zimmermann R., Hentschel C., Schrön F., Moedder D., Büttner T., Atallah P., Wegener T., Gehring T., Howitz S., Freudenberg U. (2019). High resolution bioprinting of multi-component hydrogels. Biofabrication.

[63] Sakai Y., Matsuyama Y., Takahashi K., Sato T., Hattori T., Nakashima S., Ishiguro N. (2007). New artificial nerve conduits made with photocrosslinked hyaluronic acid for peripheral nerve regeneration. Biomed. Mater. Eng..

[64] Ortuño-Lizarán I., Vilariño-Feltrer G., Martínez-Ramos C., Pradas M.M., Vallés-Lluch A. (2016). Influence of synthesis parameters on hyaluronic acid hydrogels intended as nerve conduits. Biofabrication.

[65] Hsieh F.-Y., Hsu S.-H. (2015). 3D bioprinting: A new insight into the therapeutic strategy of neural tissue regeneration. Organogenesis.

[66] Wan A.C., Mao H.Q., Wang S., Leong K.W., Ong L.K., Yu H. (2001). Fabrication of poly (phosphoester) nerve guides by immersion precipitation and the control of porosity. Biomaterials.

[67] Stang F., Fansa H., Wolf G., Keilhoff G. (2005). Collagen nerve conduits—Assessment of biocompatibility and axonal regeneration. Biomed. Mater. Eng..

[68] Klein S., Vykoukal J., Felthaus O., Dienstknecht T., Prantl L. (2016). Collagen Type I Conduits for the Regeneration of Nerve Defects. Materials.

[69] Fujimaki H., Matsumine H., Osaki H., Ueta Y., Kamei W., Shimizu M., Hashimoto K., Fujii K., Kazama T., Matsumoto T. (2019). Dedifferentiated fat cells in polyglycolic acid-collagen nerve conduits promote rat facial nerve regeneration. Regen Ther..

[70] Holliday M.A., Davison S.P. (2014). Use of polyglycolic acid nerve conduit (neurotube) to alleviate pedicle kinking in microvascular anastomosis. Plast. Reconstr. Surg..

[71] Song J., Sun B., Liu S., Chen W., Zhang Y., Wang C., Mo X., Che J., Ouyang Y., Yuan W. (2016). Polymerizing Pyrrole Coated Poly (l-lactic acid-co-ε-caprolactone) (PLCL) Conductive Nanofibrous Conduit Combined with Electric Stimulation for Long-Range Peripheral Nerve Regeneration. Front. Mol. Neurosci..

[72] Aydin T., Gurcan C., Taheri H., Yilmazer A. (2018). Graphene Based Materials in Neural Tissue Regeneration. Adv. Exp. Med. Biol..

[73] Sun Y., Liu X., George M.N., Park S., Gaihre B., Terzic A., Lu L. (2020). Enhanced Nerve Cell Proliferation and Differentiation on Electrically Conductive Scaffolds Embedded with Graphene and Carbon Nanotubes. J. Biomed. Mater. Res. A.

[74] Vijayavenkataraman S., Kannan S., Cao T., Fuh J.Y.H., Sriram G., Lu W.F. (2019). 3D-Printed PCL/PPy Conductive Scaffolds as Three-Dimensional Porous Nerve Guide Conduits (NGCs) for Peripheral Nerve Injury Repair. Front. Bioeng. Biotechnol..

[75] Jin J., Limburg S., Joshi S.K., Landman R., Park M., Zhang Q., Kim H.T., Kuo A.C. (2013). Peripheral nerve repair in rats using composite hydrogel-filled aligned nanofiber conduits with incorporated nerve growth factor. Tissue Eng. Part A.

[76] Dixon A.R., Jariwala S.H., Bilis Z., LoVerde J.R., Pasquina P., Alvarez L.M. (2018). Bridging the gap in peripheral nerve repair with 3D printed and bioprinted conduits. Biomaterials.

[77] Arcaute K., Mann B.K., Wicker R.B. (2011). Fabrication of Off-the-Shelf Multilumen Poly (Ethylene Glycol) Nerve Guidance Conduits Using Stereolithography. Tissue Eng. Part C Methods.

[78] Pateman C.J., Harding A., Glen A., Taylor C., Christmas C.R., Robinson P., Rimmer S., Boissonade F., Claeyssens F., Haycock J. (2015). Nerve guides manufactured from photocurable polymers to aid peripheral nerve repair. Biomaterials.

[79] Ma Z., Hu S., Tan J.S., Myer C., Njus N.M., Xia Z. (2013). In vitro and in vivo mechanical properties of human ulnar and median nerves. J. Biomed. Mater. Res. A.

[80] Ma X., Sun X.-L., Yang Z., Li X.-L., Ma J.-X., Zhang Y., Yuan Z.-Z. (2011). Biomechanical properties of peripheral nerve after acellular treatment. Chin. Med. J..

[81] Galarraga J.H., Kwon M.Y., Burdick J.A. (2019). 3D bioprinting via an in situ crosslinking technique towards engineering cartilage tissue. Sci. Rep..

[82] Salvatore L., Madaghiele M., Parisi C., Gatti F., Sannino A. (2014). Crosslinking of micropatterned collagen-based nerve guides to modulate the expected half-life. J. Biomed. Mater. Res. A.

[83] Du J., Jia X. (2019). Engineering nerve guidance conduits with three-dimenisonal bioprinting technology for long gap peripheral nerve regeneration. Neural Regen. Res..

[84] Maiti B., Diaz D.D. (2018). 3D Printed Polymeric Hydrogels for Nerve Regeneration. Polymers.

[85] Moldovan N.I., Hibino N., Nakayama K. (2017). Principles of the Kenzan Method for Robotic Cell Spheroid-Based Three-Dimensional Bioprinting. Tissue Eng. Part B Rev..

[86] Tomaskovic-Crook E., Crook J.M. (2020). 3D Bioprinting Electrically Conductive Bioink with Human Neural Stem Cells for Human Neural Tissues. Methods Mol. Biol..

[87] Biazar E., Heidari-Keshel S., Pouya M., Rad H., Nava M.O., Azarbakhsh M., Hooshmand S. (2013). Nanofibrous nerve conduits for repair of 30-mm-long sciatic nerve defects. Neural Regen. Res..

[88] Salaris F., Rosa A. (2019). Construction of 3D in vitro models by bioprinting human pluripotent stem cells: Challenges and opportunities. Brain Res..

[89] Tabriz A.G., A Hermida M., Leslie N.R., Shu W. (2015). Three-dimensional bioprinting of complex cell laden alginate hydrogel structures. Biofabrication.

[90] Owens C.M., Marga F., Forgacs G., Heesch C.M. (2013). Biofabrication and testing of a fully cellular nerve graft. Biofabrication.

[91] De La Vega L., Gómez D.A.R., Abelseth E., Abelseth L., Da Silva V.A., Willerth S.M. (2018). 3D Bioprinting Human Induced Pluripotent Stem Cell-Derived Neural Tissues Using a Novel Lab-on-a-Printer Technology. Appl. Sci..

[92] Miao S., Cui H., Nowicki M., Xia L., Zhou X., Lee S.-J., Zhu W., Sarkar K., Zhang Z., Zhang L.G. (2018). Stereolithographic 4D Bioprinting of Multiresponsive Architectures for Neural Engineering. Adv. Biosyst..

[93] Xu T., Gregory C.A., Molnar P., Cui X., Jalota S., Bhaduri S.B., Boland T. (2006). Viability and electrophysiology of neural cell structures generated by the inkjet printing method. Biomaterials.

[94] Vijayavenkataraman S., Yan W.-C., Lu W.F., Wang C.-H., Fuh J.Y.H. (2018). 3D bioprinting of tissues and organs for regenerative medicine. Adv. Drug Deliv. Rev..

